# Selective Photodisinfection of Bacteria and Biofilms Using Blue Light-Activated Molybdenum Nanoclusters via Dual Singlet Oxygen and Hydroxyl Radical Sensitization

**DOI:** 10.1021/acs.inorgchem.6c00945

**Published:** 2026-04-30

**Authors:** Michaela Kubáňová, Marek Dubovský, Tomáš Ruml, Kamil Lang, Jaroslav Zelenka, Kaplan Kirakci

**Affiliations:** † Department of Biochemistry and Microbiology, University of Chemistry and Technology Prague, 166 28 Praha, Czech Republic; ‡ Institute of Inorganic Chemistry of the Czech Academy of Sciences, 250 68 Husinec-Řež, Czech Republic

## Abstract

Photoinactivation of pathogens constitutes a promising strategy against the rapidly escalating antimicrobial resistance. This study highlights the water-soluble octahedral molybdenum cluster complex [Mo_6_I_8_(N_3_)_6_]^2–^ (**Mo**_**6**_) as a potent photosensitizer for antimicrobial photodynamic applications. Contrasting with previously reported molybdenum cluster complexes, **Mo**_**6**_ acts as a dual Type I/II photosensitizer, generating singlet oxygen, O_2_(^1^Δ_g_), and hydroxyl radicals even under oxygen-deficient conditions, where hydroxyl radical formation predominates. Photoinactivation was effective against Gram-positive bacteria (*Staphylococcus aureus*, *Enterococcus faecalis*), including methicillin-resistant *S. aureus* (MRSA) hospital isolate at concentrations far below mammalian cytotoxic thresholds, while *Escherichia coli* showed the expected resistance associated with its structured outer membrane. Although protein binding reduced efficacy in the presence of blood serum, the compound remained potent in serum-free conditions. The complex also achieved eradication of preformed biofilms and inhibition of biofilm formation even with varying nutrient availability. Confocal microscopy confirmed penetration of **Mo**_**6**_ into biofilm matrices and probable intracellular uptake, enabling both matrix disruption and cell inactivation. These findings highlight **Mo**_**6**_ as a stable, light-activated, and residue-free photosensitizer with strong potential for safe biofilm control and surface disinfection in hypoxic and nutrient-rich microbial habitats.

## Graphical abstract

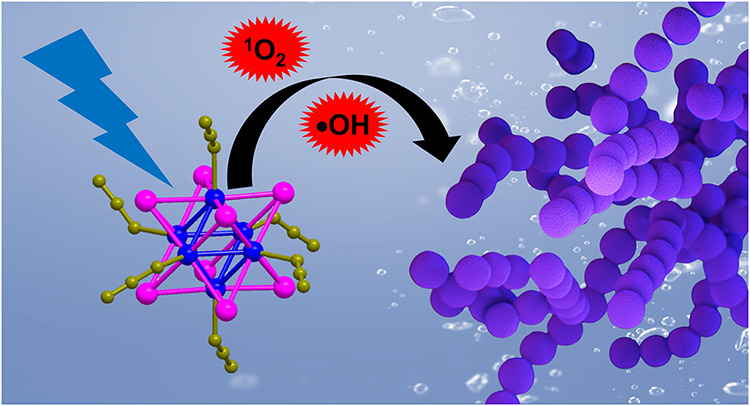

## Introduction

The rapidly escalating antimicrobial resistance threatens the global health by limiting the efficacy of established treatments for infectious diseases. Overuse and misuse of antimicrobial agents across medical, agricultural, and environmental sectors have accelerated the emergence and dissemination of multidrug-resistant pathogens, which elevate mortality rates globally.^1,2^ The transmission of resistance genes is frequently facilitated by mobile genetic elements, enabling rapid horizontal gene transfer among bacterial communities.^3,4^

Biofilms play a pivotal role in this process by providing a protective niche that enhances cell-to-cell genetic exchange and allow for persistence of resistant bacteria, especially in clinical and hospital settings.^5^ In healthcare environments, biofilm-associated pathogens substantially increase the risk of multidrug-resistant infections and complicate infection control measures, contributing to life-threatening conditions in vulnerable patient populations. With the therapeutic arsenal against multidrug-resistant bacteria severely constrained and few novel antibiotics in development, the medical community faces urgent challenges in both infection prevention and management. Given these realities, there is an imperative for alternative solutions targeting biofilm formation and eradication.^6^

Photoinactivation of pathogens has emerged as promising alternative to common chemical-based biocides and antibiotics, particularly in the context of antimicrobial resistance.^7–9^ This modality utilizes photosensitizers that are nontoxic in the absence of light and become activated upon irradiation with visible light, including blue light, leading to the generation of reactive oxygen species (ROS).^10^ Thus, produced ROS induce simultaneous damage of multiple cellular targets (membrane, proteins, DNA, etc.), thus making a development of resistance unlikely.^11^ Photoinactivation has demonstrated efficacy against Gram-positive and Gram-negative pathogens and holds significant promise for mitigating the propagation of biofilm-mediated resistance and for expanding therapeutic options in the fight against multidrug-resistant infections.^12–14^

Octahedral molybdenum cluster complexes have garnered significant attention as robust photosensitizers for photo/radiodynamic applications. These complexes, denoted [Mo_6_X_8_L_6_]*^n^*, are based on an octahedron of Mo^II^ atoms stabilized by eight strongly bonded inner ligands, typically iodine, and six labile apical ligands of either inorganic or organic origin. These cluster complexes generate high yields of singlet oxygen, O_2_(^1^Δ_g_), upon activation with X-ray, ultraviolet, and visible light up to 550 nm, making them effective type II photosensitizers.^15,16^ When compared to traditional organic counterparts such as porphyrins and phthalocyanines, these cluster complexes are less prone to photobleaching owing to their inorganic nature and do not exhibit significant self-quenching of their excited states at high concentrations or in aggregates, which could otherwise reduce their activity in biological matrices. In addition, these complexes demonstrate robust luminescence under various conditions useful for intracellular localization studies. Modification of the outer ligands allows for tuning of uptake properties and intracellular localization.^17–19^

Na_2_[Mo_6_I_8_(N_3_)_6_] (**Mo**_**6**_) (Figure 1) was among the first octahedral molybdenum cluster complexes whose biological activity was investigated.^20^ Unlike some of its later derivatives synthesized via click chemistry,^18,19,21,22^ **Mo**_**6**_ did not exhibit phototoxicity toward human cell lines. In this study, **Mo**_**6**_ is presented as a stable, water-soluble photosensitizer capable of eradicating Gram-positive bacteria in both planktonic and biofilm forms, with the aim of developing an affordable and effective surface disinfectant to prevent biofilm propagation and reduce the prevalence of resistant infections. Distinct from previously reported molybdenum cluster complexes, **Mo**_**6**_ efficiently generates not only O_2_(^1^Δ_g_) but also hydroxyl radicals under blue-light irradiation. This dual ROS production enables **Mo**_**6**_ to function as an effective photosensitizer even under oxygen-deficient conditions, where the hydroxyl radical formation predominates.

**Figure 1:**
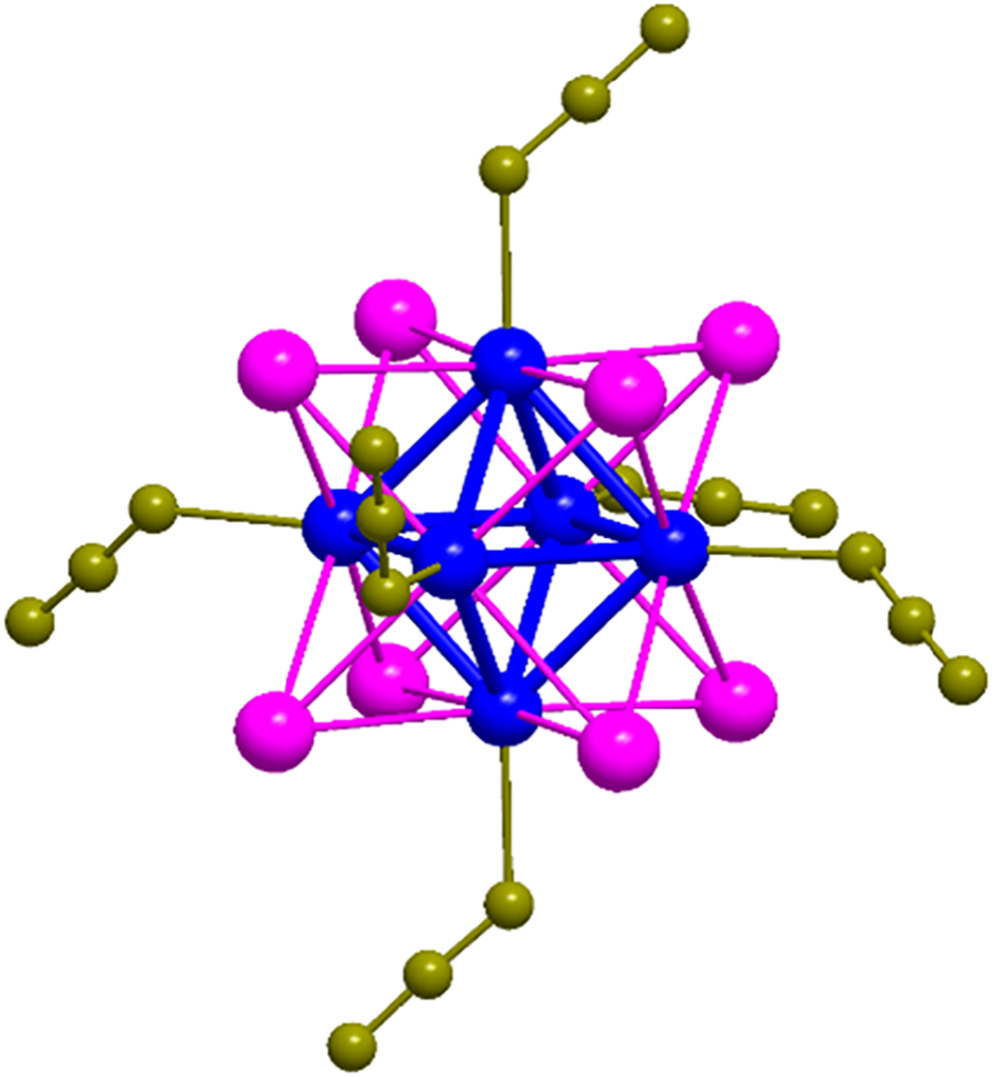
Schematic representation of Na_2_[Mo_6_I_8_(N_3_)_6_] (**Mo**_**6**_). Color codes: Mo (blue), I (magenta), N (light brown). Sodium counter-cations were omitted for clarity purposes.

## Results and Discussion

### ROS Productivity

Na_2_[Mo_6_I_8_(N_3_)_6_] (**Mo**_**6**_) is a photosensitizer of O_2_(^1^Δ_g_) with a high quantum yield of 0.65 in oxygen-saturated D_2_O as previously reported by our group.^20^ As shown in Figure 2A, measuring the weak phosphorescence of O_2_(^1^Δ_g_) centered at 1274 nm demonstrated that this feature was preserved in air-saturated water solution of **Mo**_**6**_. The assessment of the ROS production using fluorogenic dichlorodihydrofluorescein diacetate (DCF-DA) probe (Figures 2B and S1) confirmed that **Mo**_**6**_ generates ROS under 460 nm light activation. Indeed, under aerobic conditions (Figure 2B), a marked increase in fluorescence intensity relative to the control was observed, validating **Mo**_**6**_ as a photosensitizer. Importantly, significant ROS production was also detected under anaerobic conditions, demonstrating the principal finding that the compound can initiate oxidative processes even in the absence of molecular oxygen.

**Figure 2:**
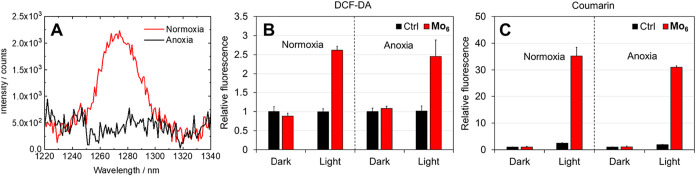
ROS production sensitized by **Mo**_**6**_ in water under aerobic and anaerobic conditions, determined via (A) measurement of O_2_(^1^Δ_g_) phosphorescence upon excitation at 400 nm, (B) use of 10 μM DCF-DA as a fluorogenic ROS probe, and (C) use of 50 μM coumarin as a fluorogenic hydroxyl radical probe. Bars marked Ctrl (black) represent control experiments performed in the absence of **Mo**_**6**_.

Based on this result, we used coumarin as a fluorogenic probe for hydroxyl radicals, the most probable ROS species formed under anaerobic conditions. Coumarin which possesses a weak fluorescence in the UV-A reacts with hydroxyl radical to form predominantly 7-hydroxycoumarin which strongly fluoresces in the blue spectral region. Irradiation of aqueous solution of coumarin in the presence of **Mo**_**6**_ led to a strong increase of the emission of 7-hydroxy coumarin when compared to the control experiment performed in the absence of **Mo**_**6**_ or in the dark (Figures 2C and S2A). Interestingly, the increase of 7-hydroxycoumarin fluorescence, when compared to the controls, was comparable in both air-saturated and deoxygenated solutions. The contribution of direct interaction between **Mo**_**6**_ and coumarin via photoinduced process was excluded as the phosphorescence lifetime of **Mo**_**6**_ in deoxygenated water was not decreased upon the addition of coumarin (Figure S2B). In addition, the use of methanol as a quencher of hydroxyl radicals confirmed its involvement in the formation of 7-hydroxy coumarin (Figure S2A).

This oxygen-independent ROS generation, most likely involving hydroxyl radicals derived from water, is a valuable property among photosensitizers and represents a key functional advantage.^23–25^ Oxygen-independent photoinactivation mechanisms have been increasingly recognized as important alternatives to conventional singlet oxygen-based pathways, particularly under hypoxic conditions.^26^ Overall, **Mo**_**6**_ constitutes a rare example of combined type I/II photosensitizer in which a proportion of excited **Mo**_**6**_ produces hydroxyl radicals while energy transfer from excited **Mo**_**6**_ in the triplet states to molecular oxygen leads to the formation of O_2_(^1^Δ_g_). This feature suggests that **Mo**_**6**_ may retain activity in hypoxic environments such as dense biofilms or confined surfaces, where antimicrobial photodynamic therapy often fails due to oxygen limitation.^27–29^

### Photoinactivation of Planktonic Bacteria

A concentration-dependent photoinactivation experiments were performed across planktonic forms of both Gram-positive and Gram-negative species (Figure 3). Notably, *Staphylococcus aureus* (Figure 3A,3C) and *Enterococcus faecalis* (Figure 3D) exhibited significant sensitivity, confirming that Gram-positive bacteria are a prime target for **Mo**_**6**_-mediated photoinactivation.^17,18,30^ Remarkably, the methicillin-resistant *S. aureus* (MRSA) strain, known for its multidrug resistance and clinical treatment difficulty (Figure 3C),^31^ showed sensitivity comparable to the model strain from the collection (Figure 3A), suggesting that the **Mo**_**6**_ effect may not be related to virulence or resistance factors. Photoinactivation of *S. aureus* by 2-logs of colony-forming units (CFU) was achieved at concentrations as low as 0.8 mg L^–1^ (0.4 μM, Figure 3A), indicating a notable activity even at submicromolar levels. Based on the exponential fit, a 3-log reduction is estimated at approximately 2.6 mg L^–1^ (∼1.3 μM) of **Mo**_**6**_. This concentration is over 2 orders of magnitude lower than the reported IC_50_ for mammalian cells, which is 185 μM for HeLa cells and 49 μM for HEK 293 cells.^20^ This large safety margin highlights the selectivity of **Mo**_**6**_ toward bacterial targets and underscores the safety potential of the compound for biomedical applications or in surface disinfection contexts without raising concerns about harmful effects on human cells, even in scenarios of incidental exposure.

**Figure 3:**
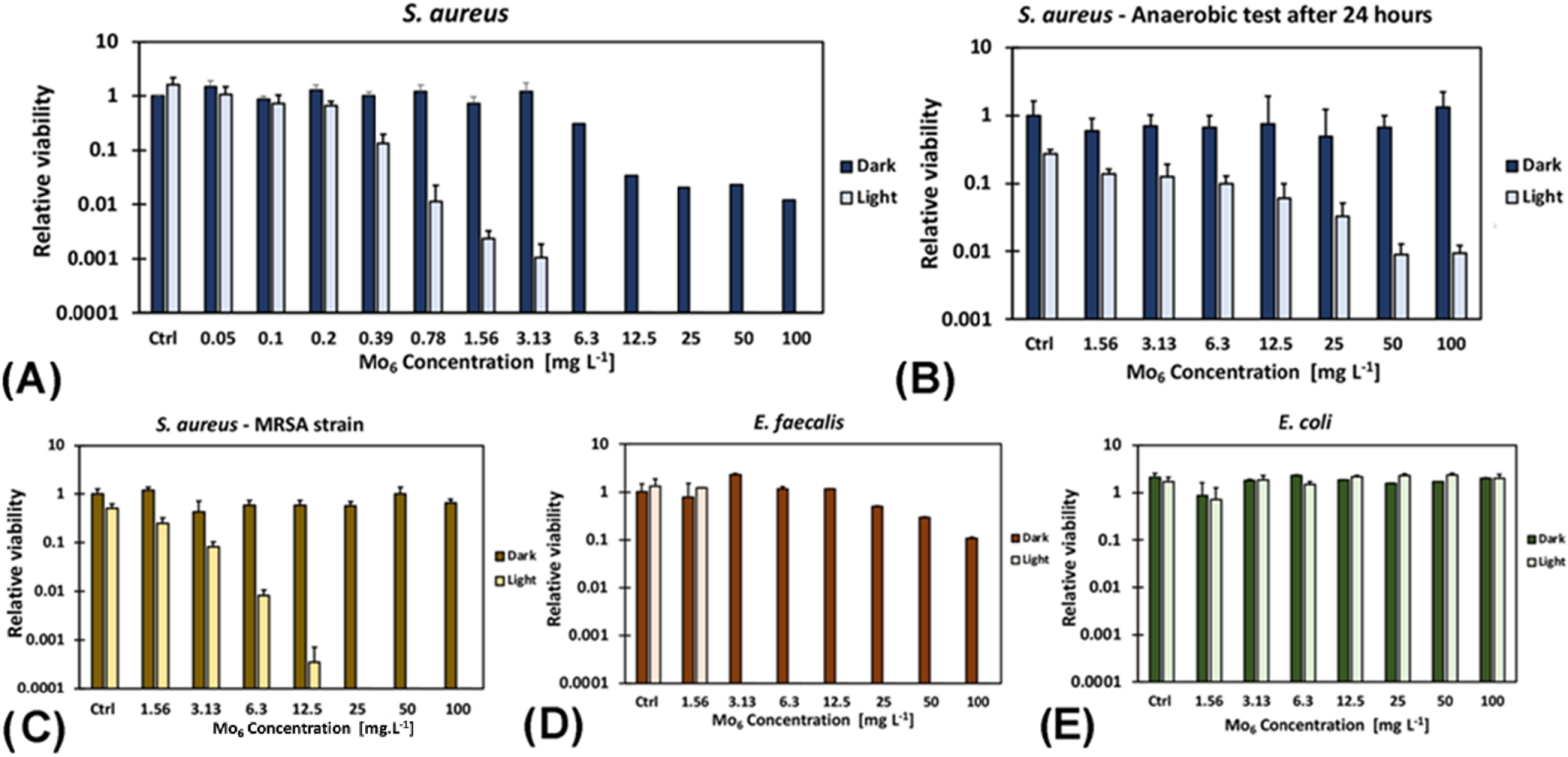
Photoinactivation of planktonic cultures upon 460 nm irradiation (18 mW cm^–2^, 15 min) in the presence of **Mo**_**6**_ in different concentrations. Control experiments (Ctrl) were performed in the absence of **Mo**_**6**_. (A) *S. aureus* (CCM 3953), (B) *S. aureus* (CCM 3953) in anaerobic conditions, (C) *S. aureus* (resistant hospital isolate), (D) *E. faecalis* (CCM 4224), (E) *E. coli* (CCM 4617**)**.

As azide anions are known antibacterial agents, the potential contribution of azide species theoretically generated from Mo_6_ through hydrolysis was also investigated. However, sodium azide at 20 mg L^–1^, corresponding to approximately 100 mg L^–1^ of fully hydrolyzed **Mo**_**6**_, did not exhibit inhibitory activity against *S. aureus* (Figure S3). Further experiments were conducted to assess the applicability of **Mo**_**6**_ as a photodynamic disinfectant. The photoinactivation efficiency was found to be independent of the solvent used for dissolving **Mo**_**6**_ powder and preparing the stock solution, whether water or dimethyl sulfoxide (DMSO) (Figure S4). Aging of the stock solution for two months resulted in a moderate reduction in the inactivation efficiency. A 2-log decrease in CFU was achieved at a concentration of 8 mg L^–1^ (4.0 μM) (Figure S5).

Notably, **Mo**_**6**_ retained substantial photoinactivation activity even after 24 h under anaerobic conditions (Figure 3B). Upon 15 min of illumination, a significant 2-log reduction in CFU was achieved at a concentration of 50 mg L^–1^. This activity is evidently associated with the hydroxyl radical formation proven above. Such oxygen-independent photodynamic activity constitutes a rare and functionally important characteristic among photosensitizers. This feature is particularly relevant for disinfection in hypoxic microenvironments, such as dense biofilms or confined surfaces, where conventional antimicrobial photodynamic therapy often exhibits reduced efficacy.^29^

High-performance photosensitizers reported in the literature are often evaluated under simplified conditions, typically in aqueous solutions or buffered systems such as phosphate-buffered saline (PBS). In contrast, the present study demonstrates that **Mo**_**6**_ retains substantial activity in more complex and biologically relevant environments, including nutrient-rich media and Eagle’s Minimal Essential Medium (EMEM) (see below). Therefore, while **Mo**_**6**_ may not represent the most optimized system in terms of maximal log reduction, its ability to function under conditions that more closely resemble real-life environments highlights its robustness and practical applicability under realistic conditions, which is increasingly emphasized in recent studies on photodynamic antimicrobial strategies.^26,32^ Importantly, it includes activity under hypoxic conditions, which are commonly encountered in biofilms and represent a major limitation for conventional photodynamic systems. Many porphyrin-, chlorin-, and phenothiazinium-based photosensitizers require micromolar to tens-of-micromolar concentrations (typically 1–20 μM, and often higher) to achieve 2–3-log reductions against Gram-positive bacteria under similar irradiation conditions.^12,14,33^ For example, chlorin and porphyrin derivatives commonly reach bactericidal effects in a low-to-mid micromolar range, while phenothiazinium dyes such as methylene blue or toluidine blue O are frequently applied at 5–50 μM.^12,34^ In contrast, **Mo**_**6**_ achieves a 2-log reduction at a submicromolar concentration, indicating higher photodynamic potency. Also, conventional disinfectants such as hydrogen peroxide or hypochlorite are typically applied at millimolar concentrations, several orders of magnitude higher than the effective **Mo**_**6**_ dose. Together, these comparisons demonstrate that **Mo**_**6**_ exhibits substantial photodynamic activity at low concentrations under relevant experimental conditions.

No significant photoinactivation was observed for the Gram-negative bacterium *Escherichia coli* (Figure 3E). This finding is consistent with previous reports indicating that Gram-negative bacteria are generally less susceptible to photodynamic inactivation, primarily due to the protective function of their outer membrane and associated efflux mechanisms, which restrict photosensitizer uptake and mitigate oxidative damage.^12^ In addition, the thick lipopolysaccharide layer of *E. coli* serves as an effective permeability barrier to many cationic and neutral photosensitizers, thereby limiting intracellular ROS generation.^35^ These structural characteristics likely account for the reduced bactericidal efficacy of **Mo**_**6**_ against *E. coli* compared with Gram-positive species.

Photoinactivation experiments conducted in EMEM without serum demonstrated that **Mo**_**6**_ retained antimicrobial activity against *S. aureus* under irradiation in a nutrient-rich environment, mimicking the human body liquids (Figure 4A). In contrast, the addition of 5% fetal bovine serum markedly suppressed phototoxicity across all tested concentrations of **Mo**_**6**_, suggesting that serum proteins interact with **Mo**_**6**_ and reduce its bioavailability for potential medicinal applications (Figure 4B). This interpretation is further supported by experiments on planktonic cells performed in the presence of varying concentrations of bovine serum albumin (BSA), the main protein of blood serum. Even low BSA levels (0.01%) significantly diminished photoinactivation, which is consistent with binding of **Mo**_**6**_ to protein (Figure S6). Such interactions are well documented for many inorganic photosensitizers and represent a major limitation for therapeutic applications, where abundant plasma proteins can rapidly attenuate photodynamic activity.^36^ Although protein binding may restrict curative or antibiotic-like use *in vivo*, the results provide important mechanistic insight into the pharmacological behavior of **Mo**_**6**_. For disinfection purposes, however, particularly on abiotic surfaces or in topical settings where serum proteins are absent, **Mo**_**6**_ retains full potency.

**Figure 4:**
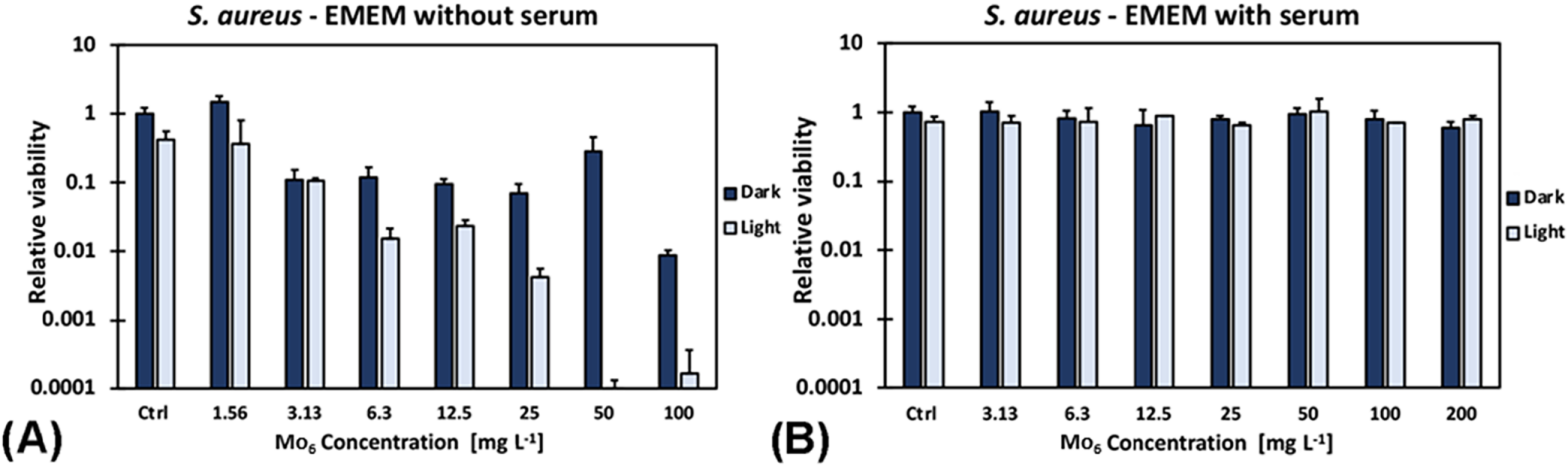
Photoinactivation of planktonic *S. aureus* under 460 nm irradiation in the presence of **Mo**_**6**_ in EMEM. (A) Without fetal bovine serum, (B) with the addition of fetal bovine serum (5%). Control experiments (Ctrl) were performed in the absence of **Mo**_**6**_.

### Photoinactivation of Bacterial Biofilms

Bacterial cells naturally occur in the biofilm form, which makes them by orders of magnitude more resistant to disinfection and antibiotics.^34^ The production of extracellular polymeric substances (EPS) varies with environmental nutrient availability and biofilm maturity. Under irradiation, **Mo**_**6**_ achieved significant biofilm eradication including for the hospital-obtained MRSA strain (Figure 5). It is remarkable that this effect was observed even with varying nutrient availability as nutrient-rich conditions promote the EPS production, thereby enhancing biofilm protection against antimicrobial treatments.^33,37^ Indeed, study on a chlorin-based photosensitizer report reduced efficacy in mature or matrix-dense biofilms.^14^ Notably, the observed eradication highlights the effective ROS-mediated activity of **Mo**_**6**_ compared to many conventional photosensitizers, which often show negligible activity against well-developed Gram-positive biofilms.^29^ These results suggest that **Mo**_**6**_ retains substantial potential for surface disinfection in complex biological environments. The high efficacy against biofilms can be attributed to the ability of **Mo**_**6**_ to generate ROS even in environments with reduced oxygen availability, such as within biofilm matrices.^27^

**Figure 5:**
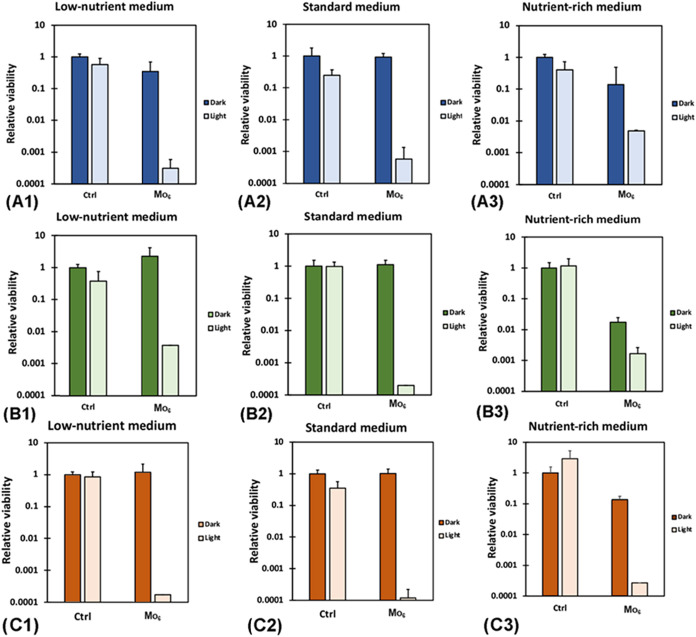
Biofilm eradication in the presence of 100 mg L^–1^ **Mo**_**6**_ in media with different abundance of nutrients. (A) *S. aureus* biofilm, (B) *E. faecalis* biofilm, (C) MRSA biofilm. (1) Peptone buffered water (low nutrients), (2) Luria–Bertani broth (medium nutrients), (3) Brain-heart infusion (high nutrients). Light-exposed sample groups were irradiated at 460 nm for 15 min, control groups (Ctrl) were incubated in the absence of **Mo**_**6**_. Some error bars are not visible due to low variability.

Prolonged irradiation in the presence of **Mo**_**6**_ not only resulted in significant eradication of planktonic bacteria in medium, but also effectively inhibited subsequent biofilm formation (Figure 6). This combined activity is particularly advantageous for preventive applications, as it demonstrates the capacity of **Mo**_**6**_ to target both established and developing biofilms, which is an enduring challenge in clinical and environmental settings.

**Figure 6:**
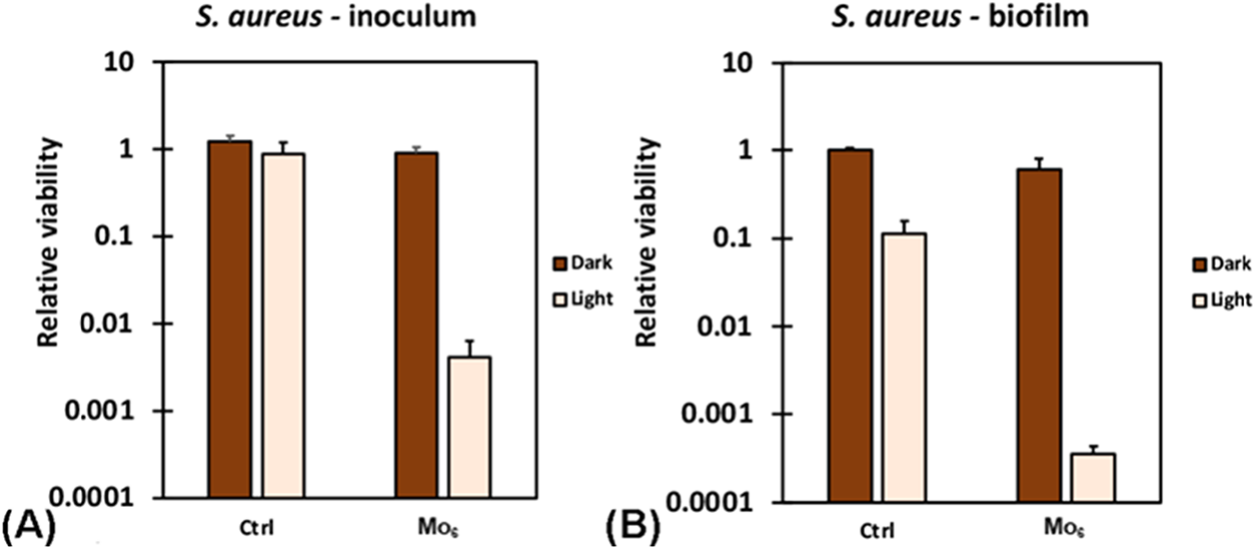
(A) Inoculum photoinactivation and (B) biofilm growth inhibition of *S. aureus* in the presence of 100 mg L^–1^ **Mo**_**6**_ in the Luria–Bertani broth medium upon 460 nm irradiation for 24 h. Control sample groups (Ctrl) were incubated in the absence of **Mo**_**6**_.

Confocal fluorescent microscopy of *S. aureus* biofilm treated with **Mo**_**6**_ revealed clear colocalization of bacterial cells (green) with **Mo**_**6**_ (red), as well as prominent **Mo**_**6**_ staining of EPS domains (large red spots), which indicate interaction of **Mo**_**6**_ with the biofilm matrix (Figure 7). Together with SYTO 9 staining of bacterial cells (Figure 7A), these observations indicate that **Mo**_**6**_ also penetrates into the biofilm layers and that its activity is not confined solely to the extracellular polymeric matrix (Figure 7C). The spatial overlap between **Mo**_**6**_ luminescence and bacterial cells further suggests intracellular uptake, implying that ROS generated upon irradiation may directly damage essential cellular components. The localization of **Mo**_**6**_ within both the biofilm matrix and bacterial cells may represent an advantage over conventional photosensitizers, which often remain sequestered in the outer matrix and consequently exhibit reduced efficacy against cells embedded in the biofilm interior.^34^

**Figure 7:**
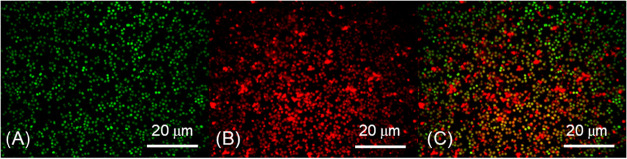
Confocal microscopy of *S. aureus* biofilm incubated with 100 mg L^–1^ **Mo**_**6**_. (A) cells stained with SYTO 9 probe excited at 488 nm and detected at 525 nm, (B) **Mo**_**6**_ luminescence excited at 405 nm and detected 600–700 nm, (C) Merged images. Scalebar 20 μm.

## Conclusions

This work demonstrates that the water-soluble **Mo**_**6**_ is an efficient photosensitizer for bacterial and biofilm photoinactivation. It generated ROS under blue-light irradiation and, importantly, retained significant activity under anaerobic conditions, via hydroxyl radical formation. This oxygen-independent ROS generation, most likely involving hydroxyl radicals derived from water, represents a key functional advantage. Unlike conventional photosensitizers that rely predominantly on singlet oxygen production and are therefore limited by oxygen availability, **Mo**_**6**_ retains activity under hypoxic conditions and expands its applicability to hypoxic environments such as dense biofilms.

Photoinactivation of planktonic bacteria was effective against Gram-positive species, including *S. aureus* and *E. faecalis*, at concentrations more than 2 orders of magnitude below reported IC_50_ values for mammalian cells, indicating a wide safety margin for use in disinfection contexts. Substantial photoinactivation was preserved under hypoxic conditions due to oxygen-independent ROS generation. Despite of the fact that *E. coli* showed marked resistance, consistent with its outer membrane barrier, the strong performance against Gram-positive strains including resistant hospital isolates underlines the robustness of the approach, which was further demonstrated by the efficient photoinhibition of biofilm formation and photoeradication of formed biofilms under varying nutrient availability.

Although protein-binding experiments revealed that serum proteins significantly reduce photoinactivation activity, thereby limiting the potential of this compound for systemic therapeutic use, its preserved efficacy in serum-free environments highlights its promise as a safe, residue-free alternative for surface disinfection. This is particularly relevant in medical and environmental settings where biofilm-associated contamination is a concern.

In contrast to chemical disinfectants such as chlorine or quaternary ammonium compounds, which act nonselectively and may trigger a resistance development, **Mo**_**6**_ offers a light-activated and residue-free mode of action, making it a promising candidate for safe surface disinfection.

## Experimental Section

### Reagents and Compounds

Na_2_[Mo_6_I_8_(N_3_)_6_] (**Mo**_**6**_) was synthesized according to procedure reported in previous publication by reacting Mo_6_I_12_ with six equivalents of sodium azide in 2-methoxyethanol at 90 °C for 3 days.^20^ Stock solutions were prepared in DMSO (10 g L^–1^, 5.3 mM) and diluted 10 times with sterile distilled water before the use, or dissolved directly in sterile distilled water (1g L^–1^, 0.53 mM) for comparison. Caution: Compound is potentially shock and temperature sensitive and should be handled with care on an appropriate scale, using personal protection precautions.

### Bacterial Strains

Opportunistic pathogenic strains *S. aureus* (CCM 3953), *E. faecalis* (CCM 4224), *E. coli* (CCM 4617) and a methicillin-resistant hospital-derived *S. aureus* isolate (Motol Hospital, Prague, Czech Republic) were used for antimicrobial testing. All strains were incubated at 37 °C for 24 h and stored at 4 °C on Luria–Bertani (LB) agar. Prior to each experiment, bacteria were freshly cultured at 37 °C for 24 h on LB agar or in LB broth.

### Photoinactivation of Planktonic Bacteria

The stock inoculum of each selected strain was prepared by diluting bacterial cultures in sterile distilled water and adjusting the suspension to a turbidity of 1 McFarland standard. Aliquots of 100 μL were mixed with 10 μL of **Mo**_**6**_ solution at the desired concentration and incubated for 2 h in the dark at laboratory temperature. For the irradiated group, samples were exposed to blue light (12 × 10 W LED source, Cameo; 460 nm, 18 mW cm^–2^) for 15 min. Bacterial inactivation was quantified using the Miles and Misra method on LB agar. Control experiments were performed using an equivalent concentration of DMSO (1% v/v), corresponding to the amount introduced by **Mo**_**6**_ addition, under both dark and irradiated conditions. All sample groups were tested in biological triplicates.

### Photoinactivation of Planktonic Bacteria in Anaerobic Conditions

The stock inoculum of each selected strain was prepared by diluting bacterial cultures in sterile distilled water and adjusting the suspension to a turbidity of 1 McFarland standard. Aliquots of 100 μL were mixed with 10 μL of **Mo**_**6**_ solution at the desired concentration and incubated for 24 h in anaerostat the dark at laboratory temperature. The anaerobic atmosphere was created using an Oxoid AnaeroGen 2.5L Sachet (Thermo Fisher Scientific). After incubation, the irradiated groups were exposed to blue light (12 × 10 W LED source, Cameo; 460 nm, 18 mW cm^–2^) for 15 min. Bacterial inactivation was quantified using the Miles and Misra method on LB agar. Control experiments were performed using an equivalent concentration of DMSO (1% v/v), corresponding to the amount introduced by **Mo**_**6**_ addition, under both dark and irradiated conditions. All sample groups were tested in biological triplicates.

### Biofilm Eradication

Bacterial biofilms were grown on coverslips in liquid medium at 37 °C for 24 h. The biofilms were then transferred into sterile distilled water and incubated with 100 mg L^–1^ **Mo**_**6**_ for 2 h in the dark at laboratory temperature., followed by either 15 min in the dark or irradiation at 460 nm (18 mW cm^–2^). Subsequently, biofilms were washed with sterile water, and coverslips were subjected to ultrasound treatment to release the biofilm into solution, which was analyzed using the Miles and Misra quantification method. Based on the nutrient availability, three types of a liquid medium were used: peptone water (low-nutrient, Millipore 77187), LB broth (medium-nutrient, Merck L3022) and BHI medium (brain-hear infusion, high-nutrient, Merck 53286). Control experiments were performed using the same concentration of DMSO (1% v/v) without **Mo**_**6**_. All sample groups were tested in biological triplicates.

### Inhibition of Biofilm Formation

Coverslips were incubated in a 1 McFarland inoculum in liquid LB medium with 100 mg L^–1^ **Mo**_**6**_ under 460 nm irradiation for 24 h (18 mW cm^–2^). The resulting biofilms were washed, sonicated in sterile water, and quantified using the Miles and Misra method. Concurrently, samples of planktonic bacteria were collected and quantified. Control experiments were conducted with the same concentration of DMSO (1% v/v) without **Mo**_**6**_. All sample groups were tested in biological triplicates.

### Interactions with Proteins

Photoinactivation of planktonic bacteria was conducted in Eagle’s Minimal Essential Medium (EMEM) with (5%) and without addition of fetal bovine serum (Merck F7524) to evaluate the influence of proteins on the Mo_6_ efficacy. In the second experiment, photoinactivation of planktonic bacteria was conducted in distilled water with 100 mg L^–1^ **Mo**_**6**_ and different concentrations of bovine serum albumin (BSA) (Millipore 81–003–3). Control experiments were conducted with the same concentration of DMSO (1% v/v) without **Mo**_**6**_. All sample groups were tested in biological triplicates.

### Confocal Microscopy

Bacterial biofilms of *S. aureus* were grown in the liquid LB medium on glass-bottom Petri dishes (MatTek) at 37 °C for 24 h. After incubation, the medium was removed, and the biofilms were gently washed with sterile distilled water. A solution of **Mo**_**6**_ (100 mg L^–1^) in phosphate-buffered saline (PBS) was added, and the samples were incubated for 2 h at room temperature in the dark. The biofilms were then stained with SYTO 9 and imaged using an Andor xD spinning disk confocal microscope mounted on an Olympus platform. **Mo**_**6**_ luminescence was excited at 405 nm and collected over 600–700 nm, and SYTO 9 fluorescence was excited at 488 nm and detected at 525 nm.

### Luminescence and ROS Production Measurements

Luminescence properties were measured on an FLS1000 spectrometer (Edinburgh Instruments, UK) using a cooled PMT-900 photon detection module (Edinburgh Instruments, UK). Singlet oxygen phosphorescence was also measured on a an FLS1000 spectrometer (Edinburgh Instruments, UK) using a Hamamatsu H10330–75 photomultiplier. Aqueous dispersions were saturated with air or argon to ensure different oxygen concentrations for luminescence analyses. The FLS1000 spectrometer was also used for time-resolved phosphorescence measurements (λ_exc_ = 405 nm, VPLED Series) and the recorded decay curves were fitted to exponential functions by the Fluoracle software (v. 2.13.2, Edinburgh Instruments, UK).

The production of ROS was determined by measuring fluorescence of dichlorodihydrofluorescein diacetate (DCF-DA) probe (10 μM) in solution of **Mo**_**6**_ in distilled water (100 mg L^–1^). Control group contained only distilled water and DCF-DA. Samples were measured before illumination and after being exposed to 460 nm light (12 × 10 W LED source, Cameo; 460 nm, 5 min, 18 mW cm^–2^). Excitation and emission wavelengths were 488 and 530 nm, respectively. Hydrogen peroxide at a final concentration of 10 μM was added as a positive control of DCF-DA oxidation and the solution was kept in the dark.

Hydroxyl radical generation was detected via its reaction with coumarin, which produces fluorescent 7-hydroxycoumarim. Air-saturated or deoxygenated dispersions of **Mo**_**6**_ (500 mg L^–1^) in deionized water containing 50 μM coumarin were irradiated with 460 nm light (12 × 10 W LED source, Cameo; 460 nm,) for 2 h. **Mo**_**6**_ was precipitated upon the addition of 500 mg of NaCl and separated by centrifugation (20,000 rpm/10 min). Excitation and emission wavelengths were 343 and 455 nm, respectively. Deionized water with 50 μM coumarin served as a control. Hydroxyl radical quenching was performed by adding 10% v/v methanol.

## Supplementary Material



## Notes

Additional experimental data (ROS production, photoinactivation of *S. aureus* under various conditions) (PDF)
