# Supplementary material for: Selective Photodisinfection of Bacteria and Biofilms Using Blue Light-Activated Molybdenum Nanoclusters via Dual Singlet Oxygen and Hydroxyl Radical Sensitization
Source: Inorg Chem. 2026 Apr 30;65(18):10205–13. doi: 10.1021/acs.inorgchem.6c00945 (PMC13169364; doi:10.1021/acs.inorgchem.6c00945)

Electronic Supplementary Information

Selective Photodisinfection of Bacteria and Biofilms Using Blue Light-Activated Molybdenum Nanoclusters *via* Dual Singlet Oxygen and Hydroxyl Radical Sensitization

Michaela Kubáňová[a,b], Marek Dubovský[b], Tomáš Ruml[a], Kamil Lang[b], Jaroslav Zelenka[a], Kaplan Kirakci[b*]*

[a]Department of Biochemistry and Microbiology, University of Chemistry and Technology Prague, 166 28 Praha, Czech Republic

[b]Institute of Inorganic Chemistry of the Czech Academy of Sciences, 250 68 Husinec-Řež, Czech Republic

Corresponding authors: Jaroslav Zelenka, zelenkaa@vscht.cz; Kaplan Kirakci, kaplan@iic.cas.cz

Content

Figure S1. Control experiments of the ROS production in water.

Figure S2. Detection of hydroxyl radicals.

Figure S3. Photoinactivation of planktonic *S. aureus* upon 460 nm irradiation in various concentrations of NaN_3.

Figure S4. Photoinactivation of *S. aureus* by $\mathbf{Mo_6}$. Stock solutions were prepared in water or DMSO.

Figure S5. Photoinactivation of *S. aureus* by 2 month-aged $\mathbf{Mo_6}$ dissolved in DMSO.

Figure S6. Photoinactivation of planktonic *S. aureus* under 460 nm irradiation in the presence of $\mathbf{Mo_6}$ and bovine serum albumin.

Figure S1. Control experiments of the ROS production in water under aerobic conditions determined with 10 μM DCF-DA as a fluorogenic ROS probe. Control experiments were performed in the absence of $\mathbf{Mo_6}$ in the dark (black), and the positive control was performed in the absence of $\mathbf{Mo_6}$ and presence of 10 μM H_2O_2 in the dark (blue).

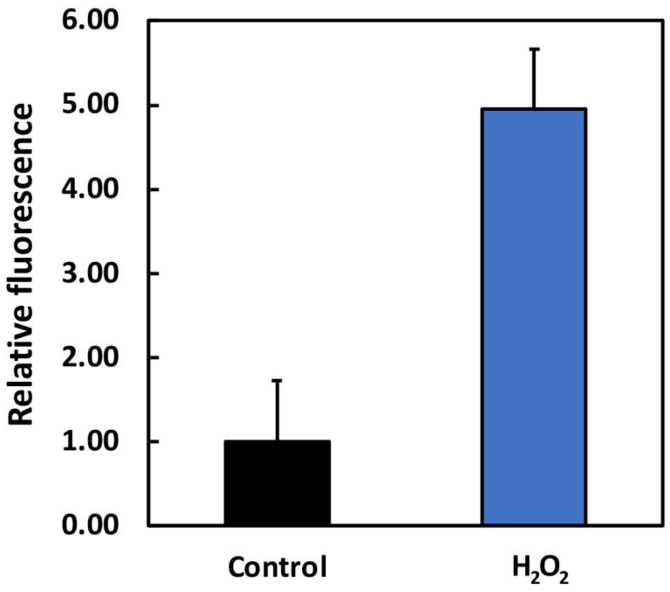

Figure S2. Detection of hydroxyl radicals. (**A**) Fluorescence spectra of 7-hydroxycoumarin after 2 h-irradiation (460 nm) of air/Ar-saturated 50 μM coumarin aqueous solutions containing $\mathbf{Mo_6}$ (0.5 mg mL^{-1}). 10% v/v methanol was used as a hydroxyl radical quencher. Excitation wavelength was 343 nm. Control experiments (Ctrl) were performed in the absence of $\mathbf{Mo_6}$ in the dark. (**B**) Phosphorescence decay kinetics of Ar-saturated water dispersions of $\mathbf{Mo_6}$ (0.5 mg mL^{-1}) in the presence and absence of 50 μM coumarin.

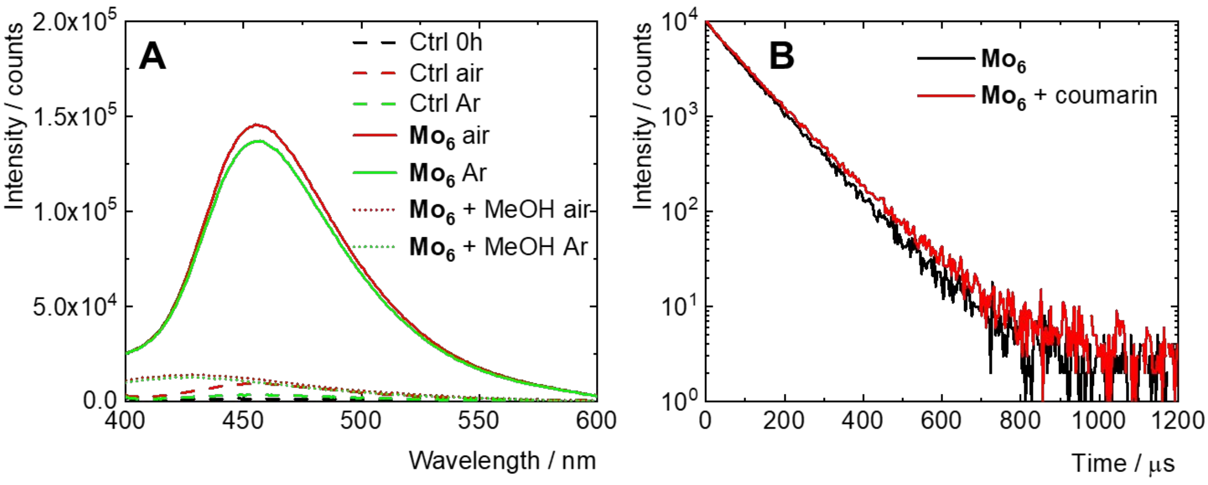

Figure S3. Photoinactivation of planktonic *S. aureus* upon 460 nm irradiation (18 mW cm^{-2}, 15 min) in various concentrations of NaN_3. Control experiments (Ctrl) were performed in the absence of NaN_3.

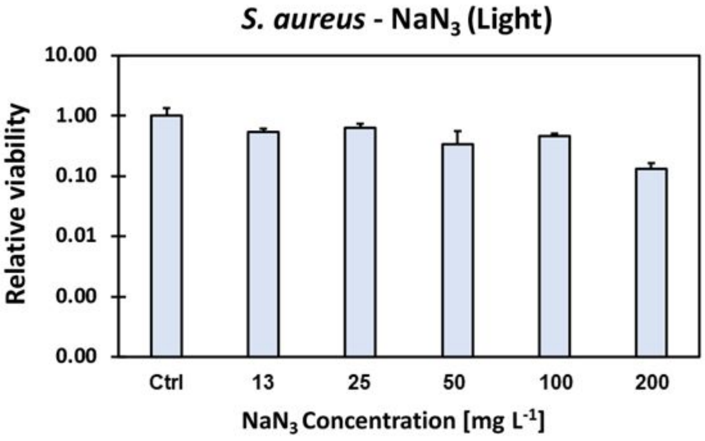

Figure S4. Photoinactivation of *S. aureus* by $\mathbf{Mo_6}$. Stock solutions of $\mathbf{Mo_6}$ were prepared in water (left) or DMSO (right). Planktonic experiments on *S. aureus* were performed upon 460 nm irradiation (18 mW cm^{-2}, 15 min) in the presence of various concentrations of $\mathbf{Mo_6}$. Control experiments were performed in the absence of $\mathbf{Mo_6}$.

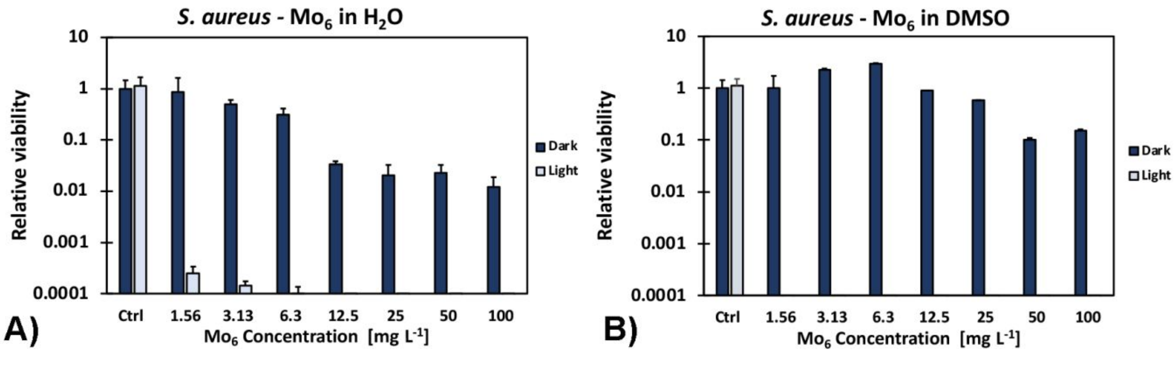

Figure S5. Photoinactivation of *S. aureus* by 2 month-aged $\mathbf{Mo_6}$ dissolved in DMSO. Planktonic experiments on *S. aureus* were performed upon 460 nm irradiation **(**18 mW cm^{-2}, 15 min**)** in the presence of different concentrations of $\mathbf{Mo_6}$. Control experiments (Ctrl) were performed in the absence of $\mathbf{Mo_6}$.

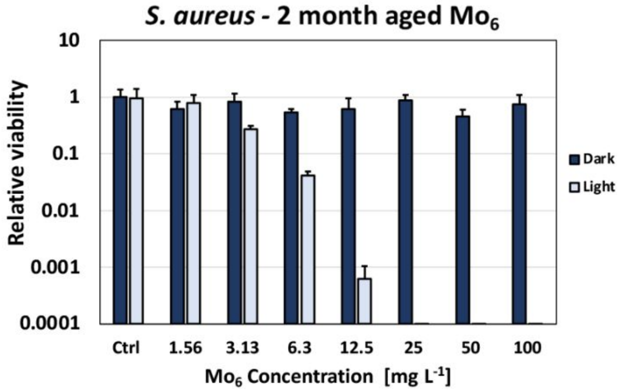

Figure S6. Photoinactivation of planktonic *S. aureus* under 460 nm irradiation **(**18 mW cm^{-2}, 15 min**)** in the presence of $\mathbf{Mo_6}$ (100 mg L^{-1}) upon the addition of bovine serum albumin (0.01 – 0.8 %). Control experiments were performed in the absence of $\mathbf{Mo_6}$.

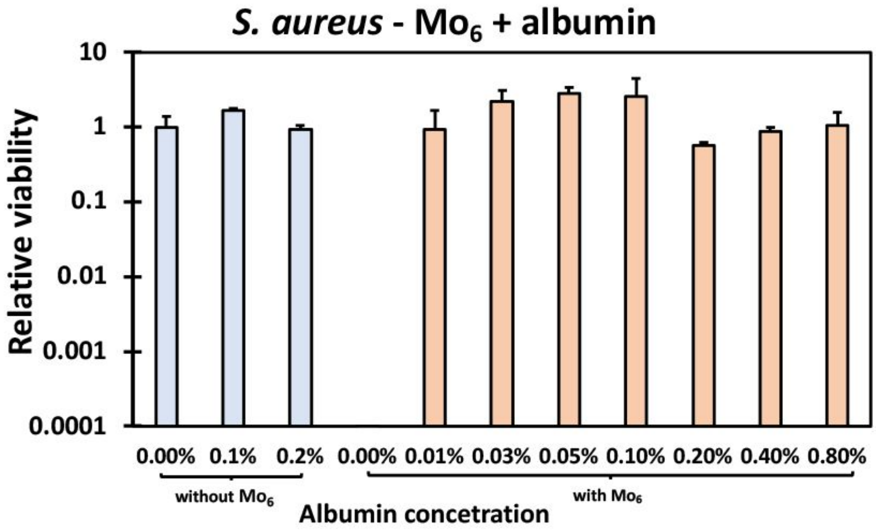

Supplement: Supplementary file 1 [file ic6c00945_si_001.pdf]
